# Disuse rescues the age-impaired adaptive response to external loading in mice

**DOI:** 10.1007/s00198-015-3142-x

**Published:** 2015-04-29

**Authors:** L. B. Meakin, P. J. Delisser, G. L. Galea, L. E. Lanyon, J. S. Price

**Affiliations:** School of Veterinary Science, University of Bristol, Langford, Bristol BS40 5DU UK

**Keywords:** Bone, Habitual activity, Mechanical loading, Neurectomy

## Abstract

**Summary:**

We aimed to determine whether aged bone’s diminished response to mechanical loading could be rescued by modulating habitual activity. By reducing background loading, aged bone’s response to loading increased to a level no different to young mice. This suggests, given the right stimulus, that ageing bone can respond to mechanical loading.

**Introduction:**

Age-related decline in bone mass has been suggested to represent an impaired ability of bone to adapt to its mechanical environment. In young mice, the tibia’s response to external mechanical loading has been shown to increase when habitual activity is reduced by sciatic neurectomy. Here we investigate if neurectomy can rescue bone’s response to loading in old mice.

**Methods:**

The effect of tibial disuse, induced by unilateral sciatic neurectomy (SN), on the adaptive response to a single peak magnitude of dynamic load-engendered mechanical strain was assessed in 19-month-old (aged) mice. In a second experiment, a range of peak loads was used to assess the load magnitude-related effects of loading on a background of disuse in young adult and aged mice. Bone architecture was analysed using micro-computed tomography (μCT) and dynamic histomorphometry.

**Results:**

In the first experiment, SN in aged mice was associated with a significant periosteal osteogenic response to loading not observed in sham-operated mice (7.98 ± 1.7 vs 1.02 ± 2.2 % increase in periosteally enclosed area, *p* < 0.05). In the second experiment, SN abrogated the expected age-related difference in the bones’ osteogenic response to peak strain magnitude (*p* > 0.05).

**Conclusions:**

These data suggest that bones’ age-related decline in osteogenic responsiveness to loading does not originate in bone cells to either assess, or appropriately respond to strain, but rather is likely to be due to inhibitory “averaging” effects derived from the habitual strains to which the bone is already adapted. If such “strain averaging” is applicable to humans, it suggests that gentle exercise may degrade the beneficially osteogenic effects of short periods of more vigorous activity.

**Electronic supplementary material:**

The online version of this article (doi:10.1007/s00198-015-3142-x) contains supplementary material, which is available to authorized users.

## Introduction

Bones ensure that their structural strength is sufficient to withstand normal loading without fracture through a process of functional adaptation. This adaptation is thought to be controlled by a local negative feedback mechanism with “target” loading-engendered strain as its objective and “off target” strains as its controlling stimulus. This homeostatic mechanism is known as the mechanostat. In old age, the function of the mechanostat appears to be impaired since despite continued functional activity, sufficient bone tissue is lost that the incidence of fragility fracture increases [1]. Several previous experimental studies suggest an impaired response to mechanical loading in aged animals including rats and mice [2–7]. In regions of cortical bone at least, the mechanism of the failure appears to be a reduced periosteal osteogenic response to loading. It has been suggested that this may be due to impaired capability of periosteal osteoblasts to proliferate [2].

Interestingly, in loading experiments in young adult mice, artificially loading the tibia in limbs where normal loading is reduced by sciatic neurectomy (SN) engenders more new bone formation than when similar artificial loading is superimposed upon the loads of normal activity [8]. In the study reported here, we aimed to investigate whether sciatic neurectomy would increase the osteogenic response to artificial loading in aged animals and perhaps restore it to that seen in young adults.

## Materials and methods

### Animals

Young adult (17-week) and aged (19-month-old) female C57BL/6 mice were obtained from Charles River Laboratories (Margate, UK). Housing and diet were as previously reported [9]. All procedures complied with the UK Animals (Scientific Procedures) Act 1986 and were approved by the institutional ethics committee.

### Ex vivo strain measurement

The strains produced by loading were calibrated in young adult and aged mice (*n* = 5) by using bonded strain gauges attached ex vivo to the medial tibial cortex at a site 37 % of the bone’s length from the proximal end [2]. Loads required to engender equivalent strains and strain rates in young and aged animals with different bone mass and architecture were calculated using linear regression as reported previously [2]. This information is presented in Supplementary Table S1. Although the load-strain relationship is affected by age, the strain distribution does not change substantially [10].

### Surgical procedures

Sciatic neurectomy (SN) was performed on the right limb as previously described [11, 12]. An incision was made caudal to the right hip joint and the biceps femoris muscle elevated to expose the nerve. This was sharply transected and a 5–7-mm segment removed. Sham surgery was performed on the right limb of control group animals. In experiment 1, (*n* = 20 aged mice) mice were weight-matched and divided evenly to sham or SN groups. In experiment 2, young and aged mice (*n* = 30 young and *n* = 30 aged mice, *n* = 5 per strain magnitude) all received SN and groups were weight-matched into even sized groups for each strain magnitude.

### In vivo external mechanical loading

Four days following SN surgery, right tibiae were subjected to external mechanical loading under isoflurane-induced anaesthesia on alternate days for eight sessions to investigate the effect of loading and SN on bone (re)modeling in young and aged mice. Left limbs were used as internal controls as previously validated [13]. The protocol for non-invasively loading the mouse tibia has been reported previously [9, 14]. A 0.5-N continuous static preload was applied in addition to which 40 cycles of dynamic load were superimposed with 10-s rest interval between each cycle. The protocol for one cycle consists of loading at a constant rate to the target peak load, hold for 0.05 s at the peak load and unloading back to the 0.5-N preload at the same rate. From the strain gage data, the peak loads required to engender strain magnitudes of 500, 1000, 1500, 2000 and 2500 με on the medial surface of the tibia at the 37 % site were calculated [2]. The strain rates during the application and release of load were 30,000 με s^−1^.

### High-resolution μCT analysis

Mice were killed 2 days after the final episode of mechanical loading. Lower legs were dissected and stored in 70 % ethanol and whole tibiae imaged using the SkyScan 1172 (Bruker, Kontich, Belgium) with a voxel size of 4.8 μm (110 μm^3^). The scanning, reconstruction and method of analysis have been previously reported [2, 9, 15]. We evaluated the effect of SN and age on changes [(right – left) / left]*100 due to loading in the trabecular region (0.25–0.75 mm distal to the proximal physis) and at the cortical site (37 % from the proximal end), according to the ASBMR guidelines [16]. The parameters measured included trabecular bone volume fraction (BV/TV), trabecular thickness (Tb.Th), trabecular number (Tb.N), trabecular pattern factor (Tb.Pf), cortical bone area (Ct.Ar), total cross-sectional area inside the periosteal envelope (Tt.Ar), medullary area (Ma.Ar), cortical thickness (Ct.Th) and bone area fraction (Ct.Ar/Tt.Ar).

### Dynamic histomorphometry

Mice were injected with calcein (50 mg/kg) and alizarin (50 mg/kg) subcutaneously on day 6 and day 14 of the loading period respectively. Following sacrifice, fixation and μCT scanning, tibiae were embedded in methylmethacrylate as reported previously [15]. Transverse sections were taken from the region in the tibia where we have previously demonstrated the response to axial loading to be maximal (37 % of the length measured from the proximal end) [13]. Images were captured using a confocal microscope with HeNe (563 nm) and diode (494 nm) lasers. Mineral apposition rates between the two labels on the endosteal and periosteal surfaces were measured in the posterio-lateral aspect of the right loaded tibiae only, where strains and bone formation engendered by loading have previously been shown to be maximal [11].

### Statistical analysis

Repeated-measures ANOVA, with post hoc least squares difference testing, was used to evaluate the effect of loading between left control and right loaded samples (loading effect) and between sham and SN groups (surgery effect) in experiment 1. The interaction “loading*surgery” was also established. The effect of SN on the interlabel distance due to loading in aged mice of experiment 1 was evaluated using an unpaired *t* test on right loaded limbs only since insufficient double label was present on left control limbs of aged mice due to their high rates of resorption. In experiment 2, linear regression analysis was used to compare the effect of age on the response to loading over a range of strain stimuli, and each line separately was compared to a gradient of zero. Values are reported as mean ± standard error of the mean (SEM).

## Results

In the first experiment, the effect of SN (*n* = 7) or sham surgery (*n* = 9) on the response of cortical and trabecular bone mass and architecture to dynamic axial tibial loading in aged C57BL/6 mice was established using μCT. There was no significant difference in body weight or tibial lengths between the groups (Supplementary Table S2), and as expected, right SN had no effect on measures of bone mass or architecture in the left control limbs between groups (Supplementary Table S2).

**Fig. 1:**
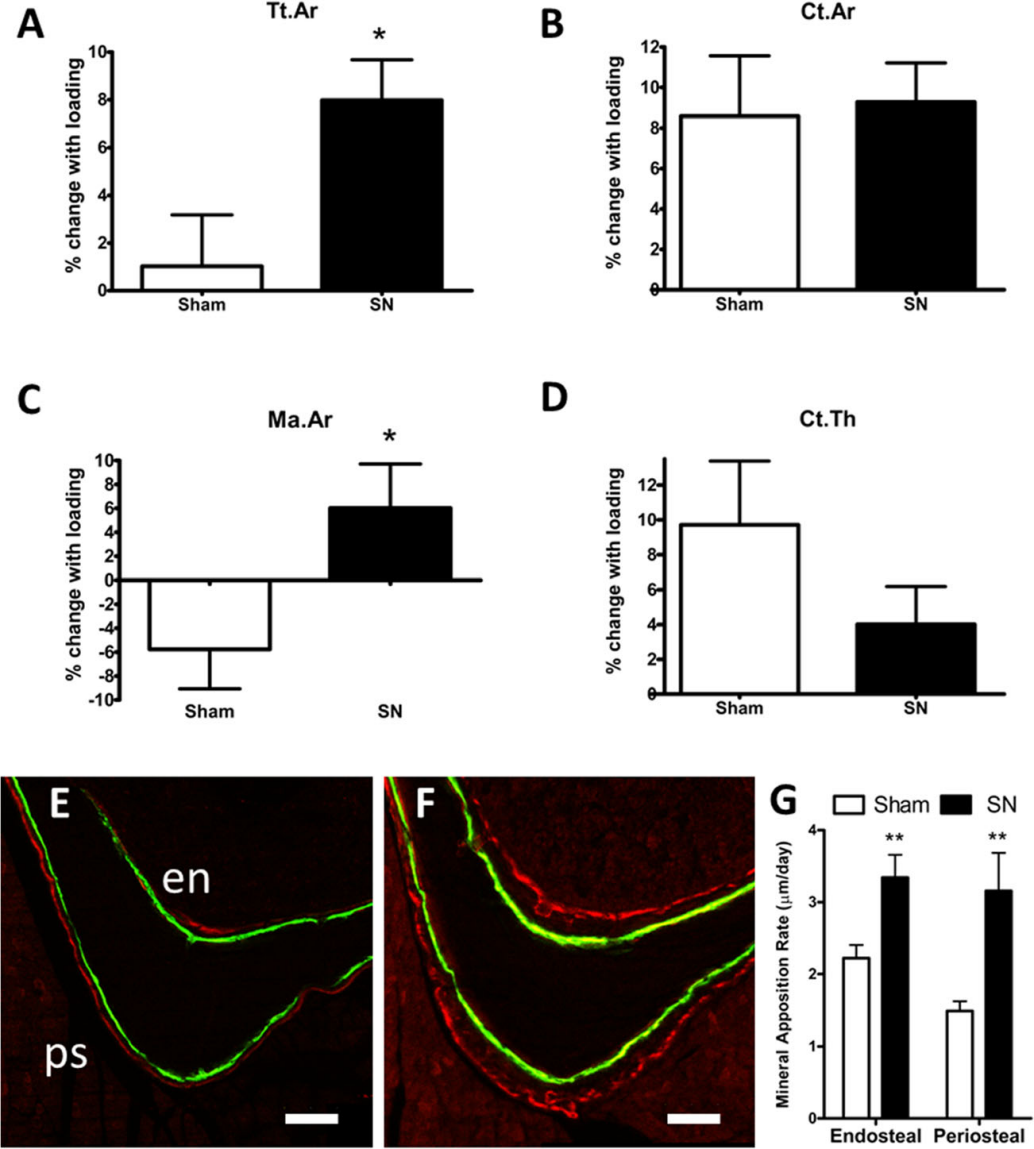
Sciatic neurectomy preceding axial tibial loading increases periosteal bone formation in aged mice. C57BL/6 female mice (19-months old) underwent right axial tibial loading sufficient to generate a strain magnitude of 2500 με following SN (*n* = 7) or sham surgery (*n* = 9). **a–d** μCT analysis showing the percentage change between left control and right loaded limbs. Data represented as mean ± SEM. **a** Total area within the periosteal envelope (Tt.Ar); **b** cortical bone area (Ct.Ar); **c** medullary area (Ma.Ar); **d** average cortical thickness (Ct.Th). **e–g** Confocal images of representative transverse sections of the posterio-lateral cortex of the tibia taken at the 37 % site illustrating calcein and alizarin flurochrome labels administered at day 6 and day 15 of loading. The *scale bars* indicate 50 μm. Endosteal surface = *en*, periosteal surface = *ps*. **e** Loaded without prior SN; **f** loaded with prior SN. **g** Mineral apposition rate at the posterio-lateral cortex. *p < 0.05; **p < 0.01

The means and SEM of the μCT parameters are presented in Supplementary Table S2. Loading significantly altered Tt.Ar, Ct.Ar, Ct.Ar/Tt.Ar, Ct.Th (*p* < 0.01 for loading as a main effect by repeated measures ANOVA) but had no effect on Ma.Ar. There was no significant main effect of surgery for any measure. Neurectomy significantly altered the effect of loading on Tt.Ar and Ma.Ar (*p* < 0.05 for the loading*surgery interaction by repeated measures ANOVA). The increase in Tt.Ar associated with loading was significantly higher in mice subjected to SN (7.98 ± 1.7 %) than the sham group (1.02 ± 2.2 %, *p* < 0.05) and the change in Ma.Ar was significantly greater in the SN group (6.02 ± 3.69) than in the sham group (−5.76 ± 3.30, *p* < 0.05). Ct.Ar, Ct.Th and Ct.Ar/Tt.Ar did not show significantly different responses to loading between surgery groups (Fig. 1 and Supplementary Table S2). This indicates that SN was associated with greater overall endosteal resorption, but concurrently greater periosteal bone formation, following loading. Confocal microscopy of the posteriolateral tibial cortex revealed an increase in the interlabel distance in SN compared to sham loaded mice on both the endosteal (+50.4 %, *p* < 0.01) and periosteal (+112.2 %, *p* < 0.01) surfaces suggesting that neurectomy is associated with both endosteal and periosteal bone formation rate at the posteriolateral cortex in response to loading (Fig. 1).

**Fig. 2:**
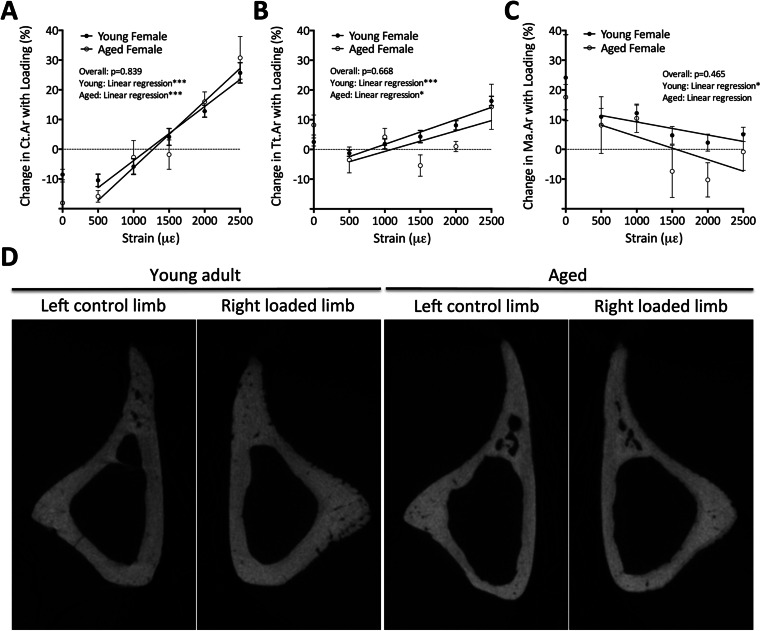
Sciatic neurectomy preceding axial tibial loading restores the response to mechanical loading in aged mice back to levels seen in young mice. Seventeen-week-old and 19-month-old aged neurectomized (SN) C57BL/6 female mice underwent right axial tibial loading sufficient to generate a range of strain magnitudes (500, 1000, 1500, 2000 and 2500 με). **a–c** μCT analysis showing the percentage change between left control and right loaded limbs in young and aged SN mice. Data represented as mean ± SEM. **a** Cortical bone area (Ct.Ar); **b** total area within the periosteal envelope (Tt.Ar); **c** medullary area (Ma.Ar). **d** Representative μCT scans from left control and right loaded young and aged SN mice. *p < 0.05; ***p < 0.001

As we have recently published [2], aged female mice have a lower periosteal osteogenic response to loading over a range of mechanical strains than young adults. We therefore proceeded to determine whether SN could restore the response to mechanical loading in aged mice back to levels seen in young mice. Linear regression analysis was used to compare the response to loading in young and aged neurectomized mice over a range of peak strains. Increases in Ct.Ar and Tt.Ar showed no significant differences between the two ages of mouse (*p* > 0.05, Fig. 2). This suggests that SN in aged mice rescued the diminished response to loading in the periosteum over the full range of peak strains investigated to the same level as that seen in young adults.

## Discussion

In this study, we first established that, as in young mice [8], the periosteal osteogenic response to short periods of dynamic loading in aged mice is greater when imposed against a background of disuse than against a background of habitual locomotor loading. We also establish that imposing a background of disuse, rather than habitual loading, eliminates any age-related difference in the osteogenic response to external loading over the full physiological range of peak strains from 500 to 2500 με. The nature of this “rescue” appears to be related to an activation of bone formation on the periosteal surface where it has previously been reported to be impaired [2, 7].

These data build upon those from the previous study which reported that in young adult mice, SN-induced disuse can increase the osteogenic response to loading [8]. These authors suggested that the nature of the rescue was due to a degree of averaging of the total strain-related stimulus. This would be consistent with the finding that the background strain stimulus engendered by habitual cage activity at the medial surface of the tibia was halved following SN from approximately 600 με in intact mice to approximately 300 με [12]. In De Souza and others’ previous study, SN was associated with an increase in both endosteal and periosteal mineral apposition rates [8]. Interestingly, in neurectomized loaded limbs of aged mice, it appears, as demonstrated by the μCT and dynamic histomorphometric imaging, that the increase in bone formation is predominantly periosteal, the same surface where the response is reported to be impaired by age [2, 7]. μCT also demonstrated an overall resorptive response endosteally (increased Ma.Ar) following SN, although this was not universal since there was a focally increased endosteal bone formation in the posteriolateral cortex. This suggests a site-specific component to the cross-sectional location of bone formation. This endosteal new bone was lamellar in appearance and occurs in the posteriolateral tibial cortex at the 37 % site, which is the same site previously identified by our group to display the greatest amount of formation following axial loading [11].

A number of previous studies have reported a lower response to mechanical loading in aged rodents [2–4, 17]. Here we show that it is possible to rescue this response by changing the character of the background strain-related environment against which the loading is imposed. Our results are consistent with those of a previous study showing that adult mice have a diminished response to loading than growing animals, but by increasing the magnitude of the strain stimulus, bone formation can be increased [5]. Taken together with our current study, these results suggest that the age-related impairment of the mechanostat is not consequent to an intrinsic failure of the mechanisms whereby bone mass increases, but a reduction in the sensitivity of these mechanisms to the strain-related stimuli to which they respond in the young, healthy skeleton. This would be consistent with our previous observation that the osteogenic response to loading in group-housed male mice was lower than that in individually housed males and in females because fighting engenders a habitual strain environment little different from that engendered by artificial loading [9]. Whether this is part of an “averaging” mechanism, as previously suggested [8], remains to be determined.

Our present study does nothing to establish the cellular mechanisms underlying the differences in the adaptive response to loading. However, it is interesting that a previous microarray study performed by our laboratory showed that loading the tibias of young adult mice in the context of disuse engendered by SN increased the number of genes differentially regulated by mechanical loading [18]. The cellular mechanisms underlying cellular mechanosensitivity remain incompletely understood, but recent work has demonstrated that mice deficient in Connexin 43 (Cx43) are resistant to bone loss seen with disuse [19] and have an improved osteogenic response to mechanical loading at the periosteal surface [20]. The authors of this study postulate that Cx43 affects regulation of bone cells’ response to mechanical loading through altering levels of cellular β-catenin signalling. Alterations in Wnt signalling at endosteal and periosteal surfaces in aged mice following disuse could underlie the primary observation noted in this study that a background of disuse is associated with a greater osteogenic response to short periods of loading than a background of habitual loading. Changes in cellular stiffness could also play a role as it has recently been suggested that reversible increases in cell stiffness engendered by mechanical stimulation may act as an intrinsic “brake” to further mechanical responses by diminishing the strain a cell experiences when the same stress is applied [21]. These in vitro findings are consistent with the well-established finding that inserting rest periods between periods of loading increases the osteogenic response to loading [3] and our present finding that “habitual” strains also blunt this response in aged mice.

In summary, the data presented here demonstrate that reducing habitual loading of the tibia in aged mice by sciatic neurectomy increases the periosteal osteogenic response to short periods of dynamic artificial loading restoring it across the full range of physiological strains to that seen in young adults. These data are consistent with the idea that the strain-related stimulus arising from long periods of normal loading may reduce the response to short periods of more osteogenic stimulation. These results also suggest that there is no inherent age-related impediment either to the accurate assessment of strain or to loading-related periosteal expansion in response to appropriate loading.

## Electronic supplementary material

Supplementary Table S1 (DOCX 44 kb)

Supplementary Table S2 (DOCX 19 kb)

Supplementary Table S3 (DOCX 44 kb)
