# Supplementary material for: Disuse rescues the age-impaired adaptive response to external loading in mice
Source: Osteoporos Int. 2015 Apr 29;26(11):2703–8. doi: 10.1007/s00198-015-3142-x (PMC4605986; doi:10.1007/s00198-015-3142-x)
Supplement: Supplementary file 1 — (DOCX 44 kb) [file 198_2015_3142_MOESM1_ESM.docx]

Supplementary Table 1

| Strain (με) | Young Female | Aged Female |
| --- | --- | --- |
| 500 | 2.99N | 2.35N |
| 1000 | 5.99N | 4.71N |
| 1500 | 8.98N | 7.06N |
| 1750 | 10.48N | 8.24N |
| 2000 | 11.97N | 9.41N |
| 2250 | 13.47N | 10.59N |
| 2500 | 14.97N | 11.77N |
| Load Rate | 500N/s | 393N/s |

Supplementary Table 1: Loading-engendered strains measured at the 37% site of the proximal tibia in representative mice were used to determine the magnitude of load required to engender strain magnitudes of 500, 1000, 1500, 1750, 2000, 2250 and 2500με in young and aged female mice. The load rate to apply an average strain rate of 30,000μεs^-1^ during loading and unloading was also calculated as previously reported (Meakin et al., 2014).
