# Supplementary material for: Disuse rescues the age-impaired adaptive response to external loading in mice
Source: Osteoporos Int. 2015 Apr 29;26(11):2703–8. doi: 10.1007/s00198-015-3142-x (PMC4605986; doi:10.1007/s00198-015-3142-x)
Supplement: Supplementary file 2 — (DOCX 19 kb) [file 198_2015_3142_MOESM2_ESM.docx]

|  | | **Sham operated (n=9)** | **Sciatic neurectomy (n=7)** |
| --- | --- | --- | --- |
| Bodyweight (g) | | 30.22 ± 4.14 | 30.28 ± 2.01 |
| Tibial length (mm) | | 18.49 ± 0.06 | 18.35 ± 0.12 |
| **Trabecular bone** | | | |
| BV/TV (%) | Left Control | 1.714 ± 0.273 | 1.572 ± 0.179 |
|  | Right Loaded | 2.166 ± 0.315 | 1.298 ± 0.281 |
| Tb.Th (mm) | Left Control | 0.044 ± 0.002 | 0.050 ± 0.001 |
|  | Right Loaded | 0.053 ± 0.003* | 0.054 ± 0.002 |
| Tb.Sp (mm) | Left Control | 0.414 ± 0.009 | 0.407 ± 0.007 |
|  | Right Loaded | 0.413 ± 0.007 | 0.438 ± 0.007* |
| Tb.N (mm^-1^) | Left Control | 0.411 ± 0.080 | 0.315 ± 0.037 |
|  | Right Loaded | 0.435 ± 0.072 | 0.234 ± 0.046 |
| **Cortical Bone** | | | |
| Ct.Ar (mm^2^) | Left Control | 0.573 ± 0.020 | 0.574 ± 0.013 |
|  | Right Loaded†† | 0.620 ± 0.021 ** | 0.628 ± 0.019 ** |
| Tt.Ar (mm^2^) | Left Control | 1.125 ± 0.022 | 1.083 ± 0.016 |
|  | Right Loaded†† | 1.137 ± 0.030 | 1.169 ± 0.022 ‡ *** |
| Ma.Ar (mm^2^) | Left Control | 0.552 ± 0.019 | 0.509 ± 0.013 |
|  | Right Loaded | 0.516 ± 0.011 | 0.541 ± 0.020 ‡ |
| Ct.Ar/Tt.Ar (%) | Left Control | 0.509 ± 0.014 | 0.530 ± 0.009 |
|  | Right Loaded†† | 0.545 ± 0.006 ** | 0.537 ± 0.014 |
| Ct.Th (mm) | Left Control | 0.117 ± 0.004 | 0.123 ± 0.003 |
|  | Right Loaded†† | 0.128 ± 0.005 ** | 0.128 ± 0.003 |
| MAR (μm/day) | Endosteal | 2.22 ± 0.19 | 3.34 ± 0.32 ^##^ |
|  | Periosteal | 1.49 ± 0.14 | 3.15 ± 0.53 ^##^ |

**Supplementary Table 1:** **Sciatic neurectomy preceding axial tibial loading increases periosteal bone formation in aged mice.** Parameters of bone mass and architecture were measured in trabecular bone of the proximal tibia (0.25-0.75mm distal to the proximal physis) and cortical bone (37% site measured from the proximal end) using µCT. Mineral apposition rate (MAR) was measured using dynamic histomorphometry. Data shown as mean ± SEM. †† = p<0.01 as a main effect for loading and ‡ = p<0.05 for the loading*surgery interaction; compared using mixed measures ANOVA. * = p<0.05, ** = p<0.01, *** = p<0.001; compared with control limb using a post-hoc pairwise comparison. ^##^ = p<0.01; compared with sham group using an unpaired t-test.
