# Supplementary material for: Disuse rescues the age-impaired adaptive response to external loading in mice
Source: Osteoporos Int. 2015 Apr 29;26(11):2703–8. doi: 10.1007/s00198-015-3142-x (PMC4605986; doi:10.1007/s00198-015-3142-x)
Supplement: Supplementary file 3 — (DOCX 44 kb) [file 198_2015_3142_MOESM3_ESM.docx]

Supplementary Table 3

| Parameter | Young Female | Aged Female | p-value |
| --- | --- | --- | --- |
| Ct.Ar | 0.839*** | 0.724*** | 0.839 |
| Tt.Ar | 0.726*** | 0.202* | 0.668 |
| Ma.Ar | 0.210* | 0.108 | 0.465 |

Supplementary Table 3: Correlation coefficients for linear regression analysis in young and aged mice with statistical comparisons. Parameters of bone mass and architecture were measured in cortical bone (37% site measured from the proximal end) using µCT at a range of strain magnitudes (500, 1000, 1500, 2000 and 2500µε). Confidence intervals were used to compare each line to zero (indicated by asterisks: * p<0.05, *** p<0.001) and to each other indicated by the ‘p-value’ column.
